# Antimicrobial Activity of Euphorbia helioscopia L. Against Methicillin-Resistant Staphylococcus aureus (MRSA) In Vitro

**DOI:** 10.7759/cureus.69840

**Published:** 2024-09-21

**Authors:** Tautvydas Ribinskas, Astra Vitkauskiene, Violeta Kareiviene, Augusta Zevzikoviene

**Affiliations:** 1 Medical School, Faculty of Medicine, Medical Academy, Lithuanian University of Health Sciences, Kaunas, LTU; 2 Department of Laboratory Medicine, Medical Academy, Lithuanian University of Health Sciences, Kaunas, LTU; 3 Department of Analytical and Toxicological Chemistry, Medical Academy, Lithuanian University of Health Sciences, Kaunas, LTU

**Keywords:** antimicrobial activity, antimicrobial resistance, bioactive compounds, euphorbia helioscopia l, mrsa, phenolic compounds

## Abstract

Background: Antimicrobial resistance is currently one of the most significant threats to medical science, necessitating the exploration of new antimicrobial agents. Methicillin-resistant *Staphylococcus aureus* (MRSA) can lead to various severe conditions such as endocarditis, meningitis, abscesses, and sepsis, and conventional antibiotics such as beta-lactams are ineffective for treating these diseases. Therefore, it is imperative to search for novel chemical substances with antimicrobial effects against MRSA. This study aimed to investigate the bioactive chemical compound extracts isolated from *Euphorbia helioscopia* L. plant material against clinical samples of MRSA collected from the Hospital of Lithuanian University of Health Sciences Kaunas Clinics.

Methodology: Bioactive compounds from the aboveground parts of *Euphorbia helioscopia *L. were isolated using methanol extraction of herbal material followed by lyophilization. The total phenolic compound concentration was determined using the Folin-Ciocalteu method. Antioxidant power was assessed through the ferric reducing antioxidant power (FRAP) assay, chemical composition was analyzed via high-performance liquid chromatography (HPLC), and antimicrobial activity against MRSA was evaluated using the disc diffusion method.

Results: The lyophilized extract of *Euphorbia helioscopia* L. exhibited antimicrobial activity against two out of five strains of MRSA. Seven phenolic compounds were identified, three of which were not previously reported as constituents of *Euphorbia helioscopia* L. More than a third of the isolated compounds were phenolic compounds.

Conclusion: *Euphorbia helioscopia* L. exhibits antimicrobial properties effective against certain strains of MRSA.

## Introduction

Antimicrobial resistance, which signifies the ability of bacteria, parasites, viruses, and fungi to resist medications, poses a significant risk of regressing to an era when common infections such as pneumonia, tuberculosis, etc. could not be effectively treated. Antimicrobial resistance is one of the top 10 threats to global health, results in 700,000 deaths per year [1], and is a significant concern to the entire medical community.

*Staphylococcus aureus* (*S. aureus*) is a Gram-positive, spheric-shaped, clustered, zoonopathogenical bacterium, which is commonly found in the nasal tract or on the skin of healthy people (about 30% of the population) [2]. It resides harmlessly on healthy skin. However, it may cause serious illnesses such as endocarditis, meningitis, abscesses, sepsis, etc. if it enters the tissues or bloodstream [3]. Methicillin-resistant *Staphylococcus aureus* (MRSA) was isolated in the early 1960s and was confirmed as the cause of the first MRSA epidemic in the United Kingdom and Denmark [4]. Based on data from the US Centers for Disease Control and Prevention (CDC), about 5% of the population carries the MRSA strains [5]. According to the WHO Global Health Observatory, MRSA has been reported to cause bloodstream infections ranging from 1.19% in Finland to 100% in Egypt and 9.8% in Lithuania [6]. MRSA is resistant to beta-lactams, including penicillin, methicillin, amoxicillin, oxacillin, and cephalosporins. In the spectrum of antibiotic resistance, medicinal plants serve as a tool for the development of new drugs. 

For more than a century, chemical compounds derived from medicinal plants have functioned as prototypes for numerous clinically validated drugs, considering that more than half of the current drugs or their derivatives are of natural origin [7]. Terpenes, alkaloids, flavones, flavanols, and phenolic compounds isolated from plants have shown positive antimicrobial activity [8]. It is indicated that phenolic compounds inhibit bacterial virulence factors, affect the plasma membrane, and suppress the formation of the biofilm. There is significant evidence that phenolic compounds have a synergistic effect with antibiotics [9-11]. Some authors argue that phenolic compounds have no adverse effect on probiotic bacteria (*Lactobacillus acidophilus* and *Lactobacillus rhamnosus*) and show higher adaptability, such as increased bacterial growth. Phenolic compounds, such as gallic acid, are metabolized into pyrogallol by gallate decarboxylase and decompose into cis-aconitate, which donates to the Krebs cycle and functions as an alternative nutrient [12-14]. Phenolic compounds possess antioxidant properties as well due to the presence of one or more aromatic rings accompanied by one or more hydroxyl groups. They can donate electron or hydrogen atoms and neutralize free radicals and reactive oxygen species [15]. This mechanism helps prevent damage to nucleic acids, proteins, and lipids and cell death [16].

*Euphorbia helioscopia* L. is a yearly herb characterized by its milky latex, and it is commonly found across Eurasia and North Africa [17]. *Euphorbia helioscopia* L. is a commonly used traditional medicine for the treatment of soft tissue swelling, rheumatic pain, cough, malaria, dysentery, scab, wart, tuberculosis, and cancer [18,19]. There are approximately 195 isolated chemical compounds, including phenolic compounds, steroids, and amino acids, which exhibit P-glycoprotein-modulating, antiproliferative, anti-inflammatory, vasodepressor, lipid-lowering, antiviral, neuroprotective, and antibacterial activities and cytotoxicity against parasites [19]. Extracts from *Euphorbia helioscopia* L. demonstrated antibacterial activity against Gram-positive bacteria, with *S. aureus* and *Pseudomonas aeruginosa* being the most susceptible bacteria [20].

The objective of this study was to investigate the antimicrobial activity of *Euphorbia helioscopia* L. extract against MRSA and explore its potential for treating diseases caused by MRSA.

## Materials and methods

Plant materials and extraction

The study utilized the aboveground part of *Euphorbia helioscopia* L. (sun spurge) from the flora of Lithuania. It was identified and collected in 2021 June from the botanist Jadvyga Balvociute organic herb farm in Venta Regional Park and supplied as dried material. The herbal material was ground into a powder and extracted using 70% methanol solution (Sigma-Aldrich, Buchs, Switzerland) in an ultrasonic bath for two hours at a temperature of 25°C, with a 1:10 ratio of herbal powder to extractant. Methanol was chosen because it showed higher effectiveness in extracting phenolic compounds and flavonoids compared to ethanolic extractants [21]. Other articles have shown that the extraction of phenolic compounds is more effective using organic solvents (such as ethanol and methanol) compared to water [22]. Then, 80% of the solvent was rotary evaporated at 25±1°C and frozen at -20°C and lyophilized, using the Beta 1-8 LSCplus lyophilizer (Martin Christ Gefriertrocknungsanlagen GmbH, Osterode am Harz, Germany) at a temperature of -50°C for 24 hours. Rotary evaporation was used for the elimination of toxic methanol, and lyophilization was employed for concentrating the extracted bioactive compounds. The lyophilized extract of herbal material was ground into powder and stored in a dry container in the dark until analysis.

The 0.1 g of lyophilized extract powder was dissolved in 10 mL of purified deionized Milli-Q® water (Millipore, Arlington, Massachusetts, United States) and sonicated for five minutes in an ultrasonic bath until all powder dissolved.

Total phenolic compound determination 

The total phenolic compound of *Euphorbia helioscopia* L. extract was determined by using the evaluated spectrophotometrical Folin-Ciocalteu reagent method by Singleton et al. [23], a determination method based on the reduction of the Folin-Ciocalteu reagent by phenolic compounds and the formation of blue complex. First, 1 mL of aqueous extract was combined with 1 mL of Folin-Ciocalteu reagent (Sigma-Aldrich, Buchs, Switzerland) and 9 mL of distilled water, ensuring thorough mixing. Then, 10 mL of 7% sodium carbonate (Sigma-Aldrich, Saint-Quentin-Fallavier, France) was added, and the mixture was diluted with distilled water to a final volume of 25 mL. The solution was incubated for 90 minutes in the dark at 20±2°C. Absorbance was measured at 750 nm using the HALO DB-20 spectrophotometer (Calamb, United Kingdom) with a blank solution. Phenolic compounds were determined using a gallic acid calibration curve. The results were expressed as the equivalent of gallic acid in mg/g of lyophilized extract dry weight.

Ferric reducing antioxidant power (FRAP) assay

The FRAP assay was determined by mixing 0.3 mol/L sodium acetate buffer (Scharlau, Sentmenat, Spain), 40 mmol/L hydrochloric acid (Fluka Chemie, Buch, Switzerland), 20 nmol/L ferric chloride (Vaseline-Fabrik Rhenania, Bonn, Germany), and 10 mmol/L 2,4,6-Tris(2-pyridyl)-s-triazine (Carl Roth, Karlsruhe, Germany) solutions. A 10 μL sample of *Euphorbia helioscopia* L. extract was thoroughly mixed with 200 μL of FRAP solution and incubated for 30 minutes at room temperature. The absorbance was measured at 593 nm using the HALO DB-20 spectrophotometer. The antioxidant power was determined using a Trolox calibration curve. The results are expressed as µmol of Trolox equivalent per gram (µmol TE/g) of lyophilized extract dry weight.

High-performance liquid chromatography (HPLC)

The qualitative and quantitative analysis of phenolic compounds was conducted using HPLC [24]. This analysis employed a Waters 2695 chromatographic system (Waters, Milford, Massachusetts, United States) equipped with a Waters 996 photodiode array and an ACE 5C18 (Advanced Chromatography Technologies, Aberdeen, Scotland) (chromatography column: 250×4.6 mm). Quantification of the compounds was performed using the external standard method. Standard solutions at a concentration of 1.0 mg/mL were appropriately diluted in methanol to create a range of concentrations for linearity studies. The regression coefficient (R^2^) for all calibration curves was 0.999. Each compound's calibration curve was constructed by plotting peak areas against their respective concentrations and analyzed using linear regression. Analytes' relative standard deviation was below 5%. The chromatographic results were processed using the Empower 2 system. Two eluents were used: (A) 0.1% trifluoroacetic acid (Sigma-Aldrich, Steinheim, Germany) and (B) 100% acetonitrile (Sigma-Aldrich, Steinheim, Germany). The elution followed the protocol outlined in Table 1. The injection volume was 10 µL, with a mobile phase flow rate of 1 mL/min, an elution duration time of 81 minutes, and a column temperature maintained at 25°C. The phenolic compounds in *Euphorbia helioscopia* L. extract were identified based on retention time and UV absorption analysis from 300 to 360 nm. The extract of *Euphorbia helioscopia* L. was diluted 10 times with 70% ethanol (v/v).

**Table 1:** HPLC elution scheme. Eluent A: 0.1% trifluoroacetic acid. Eluent B: 100% acetonitrile. HPLC: high-performance liquid chromatography

Time (minutes)	A (%)	B (%)
0	95	5
8	85	15
30	80	20
48	60	40
58	50	50
65	50	50
66	5	95
70	5	95
71	95	5

Antimicrobial activity against MRSA

In this research, MRSA strains (1; 20927; 21348; 22039; 22958) were isolated from clinical samples collected from the Hospital of Lithuanian University of Health Sciences Kaunas Clinics for examination. The samples were taken from the Lithuanian University of Health Sciences Kaunas Clinics Department of Laboratory Diagnostics Clinics collection. The MRSA strain number represents the collection number. The minimum inhibitory concentration (MIC) of *Euphorbia helioscopia* L. for inhibiting MRSA growth was determined using the disc diffusion method. Sterile disks with a diameter of 6 mm were used for the study. Mueller-Hinton agar plates were inoculated with 0.5 McFarland standard solutions of MRSA, and sterile discs were placed on the agar and impregnated with 10 µL, 25 µL, 50 µL, and 75 µL prepared extracts of *Euphorbia helioscopia* L. (0.1 g lyophilized extract in 1 mL distilled water). Impregnation with volumes greater than 25 µL was carried out in several stages, using 25 µL of the solution each. Distilled water was used for the blank test. The cultures were incubated for 24 hours at 35±1°C. All tests for antimicrobial activity were repeated three times.

## Results

Total phenolic compound determination

The total phenolic compound in the *Euphorbia helioscopia* L. lyophilized extract, determined using the Folin-Ciocalteu reagent, was 391.10±4.38 mg/g of gallic acid equivalent (GAE) by lyophilized extract mass (mg GAE/g±SD, n=3).

FRAP assay

The FRAP in the lyophilized extract of *Euphorbia helioscopia* L. was 1037.5±26.4 µmol TE/g based on the Trolox (µmol TE/g±SD, n=3).

HPLC

In the study, the HPLC method was used for the qualitative and quantitative analysis of the dry lyophilized extract of *Euphorbia helioscopia* L. In the extract, gallic acid, neochlorogenic acid, chlorogenic acid, ellagic acid, hyperoside, quercetin compound, and quercetin were identified. The highest concentrations were determined of neochlorogenic acid, ellagic acid, hyperoside, and quercetin compound. Table 2 presents the concentrations of compounds in *Euphorbia helioscopia* L. lyophilized extract.

**Table 2:** Concentrations of compounds in Euphorbia helioscopia L. lyophilized extract.

Compound	Concentration (μg/g)
Gallic acid	949
Neochlorogenic acid	2702
Chlorogenic acid	1938
Ellagic acid	2414
Quercetin	41
Hyperoside	5344
Quercetin compound	8207

Antimicrobial activity against MRSA

**Figure 1:**
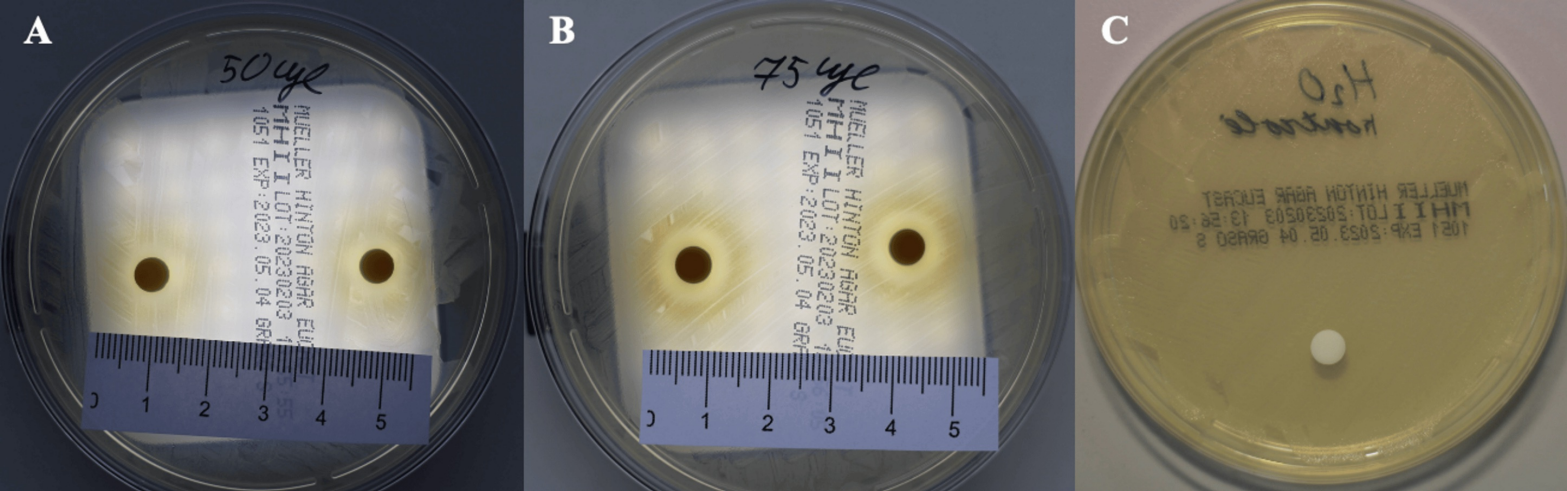
MRSA strain no. 1 inhibition zones were determined by the disc diffusion method using 50 µL (A) and 75 µL (B) of Euphorbia helioscopia L. lyophilized extract, with distilled water (C) as the control group. MRSA: methicillin-resistant *Staphylococcus aureus*

The antimicrobial activity of *Euphorbia helioscopia* L. lyophilized extract was tested against five MRSA strains. Impregnation with 10 µL and 25 µL of the extract showed no antimicrobial effect on any of the strains. Table 3 presents the antimicrobial activity for MRSA using 10 µL, 25 µL, 50 µL, and 75 µL of the extract. The highest antimicrobial activity was observed in strain no. 1 (Figure 1).

**Table 3:** MRSA inhibition zones determined by the disc diffusion method using 10 µL, 25 µL, 50 µL, and 75 µL Euphorbia helioscopia L. lyophilized extract (mean±SD, n=3). ND: no detected activity; MRSA: methicillin-resistant *Staphylococcus aureus*

Strain no.	Inhibition zone (mm)			
	10 µL	25 µL	50 µL	75 µL
1	ND	ND	10±0	12±0
20927	ND	ND	ND	ND
21348	ND	ND	ND	8±0
22039	ND	ND	ND	ND
22958	ND	ND	ND	ND

## Discussion

In the overview of *Euphorbia helioscopia* L. by Yang et al., 195 chemical compounds were isolated, with 15 of them being flavonoids. There is evidence that quercetin has anti-inflammatory activity and gallic acid has antiproliferative activity [19]. Neochlorogenic acid, ellagic acid, and hyperoside were not mentioned as being detected in *Euphorbia helioscopia* L. In their article, Chen et al. noted that phenolic compounds act as antimicrobial agents by damaging the bacterial cell wall, disrupting cell membrane structure and function, inhibiting macromolecule synthesis, disturbing energy metabolism, affecting biofilm formation, influencing quorum sensing systems, and impacting the cyclic di-GMP system [25]. There is evidence suggesting that phenolic compounds exhibit a synergistic effect with antibiotics. Quercetin, identified in *Euphorbia helioscopia* L., demonstrates antimicrobial activity against clinical strains of MRSA, thereby enhancing the efficacy of rifampicin and ciprofloxacin. The authors of the article propose that quercetin's mechanism of action involves inhibiting the activity of bacterial topoisomerases [26-28]. Given the established synergistic activity of quercetin, it is a valid option to further investigate the antimicrobial activity of *Euphorbia helioscopia* L. extracts in combination with commonly used antibiotics. Therefore, information regarding the antimicrobial activity of phenolic compounds will enable the reduction of effective drug concentrations used in the treatment of infectious diseases caused by MRSA. It is important to mention that the antimicrobial assays of phenolic compounds were conducted solely in vitro and their effects in vivo remain unknown [29]. In the research by Zhu et al., it was found that the essential oil of *Euphorbia helioscopia* L. exhibits antimicrobial activity against *Staphylococcus aureus*,* Enterococcus faecalis*, *Escherichia coli*, *Shigella dysenteriae*, and *Candida albicans* fungi [30]. Lone et al. proved that methanolic and aqueous extracts of *Euphorbia helioscopia* L. additionally have antimicrobial activity against *Klebsiella pneumoniae*,* Pseudomonas multocida*, and *Aspergillus flavus* [31]. That proves that the use of *Euphorbia helioscopia* L. as an antimicrobial agent has great prospects.

Previous studies have indicated that the extraction methodology of bioactive phenolic compounds from *Euphorbia helioscopia* L. herbal material yields significant results in total phenolic compound concentration. Mustafa et al. demonstrated that stirring and mixing the ground material of *Euphorbia helioscopia* L. in solvents, followed by rotary evaporation at 40°C and proper drying in a Petri dish at the same temperature, resulted in lower concentrations of total phenolic compounds. Specifically, the total phenolic compound concentration was found to be 16 times lower when methanol was used as the solvent, 35 times lower with ethanol, and 84 times lower with water, compared to the methanol extraction and lyophilization method employed in this study [32]. The ultrasound-assisted extraction method is not expensive, and neither temperature nor solvent-to-sample ratio influences the concentration of total phenolic compounds [33]. The research by ElNaker et al. demonstrated that oven-drying at 40°C could be as effective as lyophilization in preserving pharmacologically active compounds. Therefore, lyophilization is a much better choice for aqueous extracts [34]. In the context of the potential medical applications of *Euphorbia helioscopia* L., water solutions are in a better position.

The antioxidative power of *Euphorbia helioscopia* L. was proved by this research. The other authors' publications prove that *Euphorbia helioscopia* L. antioxidative power correlates with total phenolic compound concentration; therefore, the correlation is visible in different parts of the plant [35,36]. Mustafa et al. determined the FRAP assay of *Euphorbia helioscopia* L. extracts using ferrous sulfate for the expression of the assay. The study showed the highest FRAP assay value in methanolic extract 758.90±25.21 µmol Fe^2+^/g [32]. This suggests that the lyophilization method may be more effective in extracting the compounds responsible for antioxidant power. The research conducted by Saleem et al. demonstrated the positive effects of *Euphorbia helioscopia* L. extracts when administered in vivo. Both latex and leaf extracts were orally administered to mice daily for two weeks. The results indicated a significant increase in the levels of antioxidant enzymes such as catalase, superoxide dismutase, and glutathione. The study revealed that the extracts inhibited the generation of free radicals, thereby minimizing their effects on liver and kidney function, lipid levels, and the complete blood count in mice [37]. That information on *Euphorbia helioscopia* L. antioxidative power helps us to comprehend the broader potential of *Euphorbia helioscopia* L. usage in the additional treatment of diseases caused by free radicals and to find the best method for extracting bioactive compounds.

Study limitations

A potential limitation of this research is that it did not test which specific phenolic compound affects antimicrobial activity or whether it is the combination of phenolic compounds that impacts MRSA. Only a small number of MRSA strains were tested. Testing more strains would allow for broader conclusions about the effect of *Euphorbia helioscopia* L. extract on MRSA. Additionally, the combination of *Euphorbia helioscopia* L. extract with common antibiotics was not tested, which could potentially yield significant results and provide a wider opportunity for using *Euphorbia helioscopia* L. extracts in disease treatment.

## Conclusions

The investigation revealed that *Euphorbia helioscopia* L. exhibits antimicrobial activity against two out of five MRSA strains obtained from clinical specimens. Phenolic compounds, comprising more than a third of the extract's constituents, were identified as potential contributors to its antimicrobial effect. Notably, neochlorogenic acid, ellagic acid, hyperoside, and quercetin compound were found in substantial quantities within the extract. Significant antioxidant activity was observed in *Euphorbia helioscopia* L., indicating a potentially beneficial impact on human health. Consequently,* Euphorbia helioscopia* L. may serve as a valuable resource in combating MRSA infections or in the development of novel therapeutic agents.
